# Editorial: Behavioral Immune System: Its Psychological Bases and Functions

**DOI:** 10.3389/fpsyg.2021.659975

**Published:** 2021-02-26

**Authors:** Kazunori Iwasa, Yuki Yamada, Tsunehiko Tanaka

**Affiliations:** ^1^Department of Educational Psychology, Faculty of Education, Shujitsu University, Okayama, Japan; ^2^Faculty of Arts and Science, Kyushu University, Fukuoka, Japan; ^3^Educational Psychology Course, Faculty of Education, Niigata University, Niigata, Japan

**Keywords:** behavioral immune system, disgust, disease avoidance, emotion, cognition, perception, attitudes

Currently, the world is in the midst of the COVID-19 pandemic. This has reminded us of the threat posed by infectious diseases. In some countries, cities were temporarily locked down and people were restricted from traveling. This severely affected our economy and culture, and mental health problems associated with this situation have arisen. This infectious disease has significantly impacted societies and people. Moreover, the threat has significantly altered individual behavior. People have become socially distant and have had to frequently sterilize their hands. In some areas, wearing masks has become mandatory, and there have even been legal penalties for those who violated local rules. The pandemic has changed our behavior dramatically.

These behavioral changes, both at the individual and community levels, appear to have been driven by the goal of disease avoidance. From the standpoint of this Research Topic, it can also be said that the threat of infectious diseases has resulted in a collective activation of the behavioral immune system (BIS). When we started this Research Topic, we did not anticipate this situation. Now, however, it has become highly relevant. While not welcome, it has provided a basis for understanding human behaviors under pandemics.

BIS is a motivational system with the goal of disease avoidance. It estimates the presence of pathogens from perceptual cues in the environment and elicits relevant emotional and cognitive responses. Such responses induce avoidance behavior in a pathogenic environment (Schaller and Park, 2011). This sequence of psychological responses, by preventing contact with and penetration into the body of these infectious sources, compensates for the physiological immune system which can sometimes be physically high cost (Murray and Schaller, 2016). The theory of BIS has an evolutionary psychological basis, and it has been used to explain and predict a wide range of human behaviors (Ackerman et al., 2018). Additionally, the description of detailed mechanisms for disease avoidance redefined the adaptive value of disgust, which is a key emotion in BIS.

BIS has been revealed to be associated with diverse human behaviors. However, it remains unclear what components it consists of and how it is derived from our biological foundations. In this regard, Murray et al. provided a comprehensive discussion of the psychophysiological basis of BIS, which included sensory, cellular, and genetic perspectives. They offered an in-depth description of the current state of PsychoBehavioroimmunology regarding BIS, including an extensive review. The work of Cañas-Gonzaléz et al. demonstrated that physiological immunity affects the state of depression. A study by Iwasa et al., which provided a psychophysical analysis of visual pathogen detection, can be understood as a practical example of a specific study for the general remarks made by Murray et al.. Additionally, Shakhar provided a conceptual analysis of a more inclusive view of BIS based on its genetic origins. While referring to Hamilton (1964) inclusive selection theory, Shakhar stated that BIS works to protect not only an individual itself but also the kin around the person. In this regard, BIS protects others through the individual's disease behaviors and social immunity behaviors, favoring the whole “kin selection.” This is an attempt to conceptually extend BIS and provide a fresh perspective in this field.

The study of the relationship between BIS and various human behaviors elucidates its functional characteristics. One of the human behaviors affected by BIS is sexual conduct. Sexual behavior is inevitably associated with the risk of sexually transmitted infections (STIs). Sexual arousal and physical attractiveness influence male sexual decision making; considering the risk of STI, the disgust emotion may also be associated with it. Oaten et al., using a survey to detect substantial sexual arousal, indicated that arousal decreases state disgust and STI risk judgments, and increases willingness to have sex. Furthermore, they identified that low trait disgust predicted a strong willingness to have sex. This study is a good example of the functional characteristics of BIS, describing how the sexual motivation system and BIS work against each other to control sexual decision-making.

Considering the functional aspects of BIS, we cannot ignore its pervasive influence on our attitudes. Liuzza et al. revealed that moral judgments about purity are influenced by disgust sensitivity to body odor; Tsegmed et al. observed that negative implicit attitudes toward agricultural and aquatic products from Fukushima were related to thoughts about nuclear contamination. These studies reiterate how the BIS functions to avoid disease through attitude change. The work of Stewart et al., revealing the impact of disgust on people's religiosity, is another example depicting the influence of BIS on people's attitudes. In contrast, some articles presented new research agendas in this area. Horita and Takezawa reexamined the impact of pathogen stress on collectivism and conformity using Bayesian statistics and revealed that the impact may be more limited than originally thought. Wu et al. revealed that the degree of acceptance of ingroup members tended to decrease compared to outgroup members in the context of disease (e.g., ingroup derogation). Concerning the association between BIS and outgroup prejudice, Kusche and Barker's article, which proposed a model including social contexts such as family environment and mass media, provided us with substantial inspiration.

Research on BIS has come to encompass a wide range of human behavior. Ito et al. discussed the role of BIS in social anxiety in terms of the behavioral inhibition system and behavioral activation system; the impact of BIS on mental and physical health is one of the areas that is expected to grow, especially in today's world, under the influence of serious infectious diseases. In conclusion, based on the psychophysiological foundation of BIS, it is necessary to further clarify the relationship between perception, cognition, personality, social relationships, and psychiatric disorders, and individual behavior and attitudes, thus developing a conceptual, mathematical, and psychological model that comprehensively explains their functioning. Only then will we be able to understand the practical applicability of BIS to psychotherapies and policymaking.

## Author Contributions

The editorial was drafted by KI and YY and approved by the topic co-editors. All authors listed have made a substantial contribution to this Research Topic and have approved this editorial for publication.

## Conflict of Interest

The authors declare that the research was conducted in the absence of any commercial or financial relationships that could be construed as a potential conflict of interest.
